# Fatal Meningitis from Shiga Toxin–Producing *Escherichia coli* in 2 Full-Term Neonates, France

**DOI:** 10.3201/eid2908.230169

**Published:** 2023-08

**Authors:** Guillaume Geslain, Aurélie Cointe, Philippe Bidet, Céline Courroux, Soumeth Abasse, Patricia Mariani, Stéphane Bonacorsi

**Affiliations:** Robert Debré University Hospital Assistance Publique-Hôpitaux de Paris, Paris, France (G. Geslain, A. Cointe, P. Bidet, C. Courroux, P. Mariani, S. Bonacorsi);; Université Paris Cité INSERM, Paris (G. Geslain, A. Cointe, P. Bidet, S. Bonacorsi);; Mayotte Hospital, Mamoudzou, Mayotte Island, France (S. Abasse)

**Keywords:** Shiga toxin–producing *Escherichia coli*, Shiga toxin, *Escherichia coli*, neonate, newborn, bacteria, enteric infections, meningitis/encephalitis, France

## Abstract

We report fatal meningitis in 2 neonates in France caused by Shiga toxin 1–producing *Escherichia coli*. Virulence factors capsular K1 antigen and salmochelin were present in both strains, potentially representing a new hybrid pathotype. Clinicians should remain aware of emerging pathotypes and design therapeutic strategies for neonatal *E. coli* infections.

*Escherichia coli* can acquire virulence factors associated with increased pathogenicity, causing intestinal or extraintestinal infections. Shiga toxin (Stx)–producing *E. coli* (STEC) cause intestinal infections and hemolytic uremic syndrome (HUS). *E. coli* virulence factors K1 antigen and salmochelin are associated with neonatal *E*. *coli* meningitis (*1*). Hybrid pathotypes have been described, such as Stx-producing O80:H2, which causes HUS and bacteremia because of extraintestinal virulence–associated plasmid pS88 (*2*). We report 2 cases of full-term neonates who died of meningitis caused by STEC serotypes O117:H7 and O156:H7.

Patient 1, an 8-day-old full-term boy born in the Paris, France, area, was seen at the emergency department because of a 48-hour history of abdominal pain and rectorrhagia without fever and drowsiness since that morning. Septic shock with colitis developed rapidly, requiring admission to the pediatric intensive care unit (PICU). Hemolysis was not observed, and initial acute kidney failure resolved promptly. Blood culture results were positive for *E. coli* K1, urine culture results were negative, and 3 stool cultures yielded STEC. Cerebral magnetic resonance imaging on PICU day 7 showed severe diffuse lesions indicating meningoencephalitis and subdural empyema. Cerebrospinal fluid (CSF) obtained by lumbar puncture 2 days later contained 2,270 leukocytes/mm^3^, showed negative Gram stain and culture, and was PCR-positive for K1 and β-glucuronidase genes, indicating *E. coli* meningitis. Prompted by clinical and laboratory features potentially associated with HUS, PCR on blood and CSF DNA were performed and identified *stx1*. The patient died on day 28.

Patient 2, a full-term boy born in an overseas department of France, was transferred to a PICU at 4 days of age because of fever, drowsiness, and failure to feed. Septic shock without colitis developed rapidly, without hemolysis or initial thrombocytopenia. On PICU day 4, blood culture results were positive and urine culture results negative for *E. coli*; CSF contained 900 leukocytes/mm^3^ and showed *E. coli* growth in culture; Stx was not initially evaluated. The patient died on PICU day 9.

Whole-genome sequencing of blood and stool isolates (patient 1) and CSF isolate (patient 2) indicated the *E*. *coli* strains belonged to sequence type (ST)504 and phylogroup B2; serotypes were O117:H7 for patient 1 and O156:H7 for patient 2. Both strains harbored the gene encoding Stx1a protein but not *eae* (intimin) or *ehxA* (enterohemolysin) genes (*3*,*4*). Genetic determinants for extraintestinal virulence factors K1 capsular antigen, yersiniabactin, and salmochelin were present in both strains and the aerobactin operon in the O117:H7 strain. Screening for confirmed or putative virulence factors as previously described (*5*) (Table) showed neither strain harbored an pS88-like plasmid (*6*).

**Table:** Genes encoding confirmed or putative virulence factors found in Shiga toxin–producing *Escherichia coli* ST504 strains that caused fatal meningitis in 2 full-term neonates, France*

Gene	Gene function	*E. coli* O117:H7	*E. coli* O156:H7
*iucC*	Aerobactin biosynthesis	Positive	Negative
*iutA*	Aerobactin receptor	Positive	Negative
*fyuA*	Yersiniabactin receptor	Positive	Positive
*irp2*	Yersiniabactin biosynthesis	Positive	Positive
*iroN*	Salmochelin receptor	Positive	Positive
*iroD*	Salmochelin biosynthesis	Positive	Positive
*chuA*	Hemin uptake	Negative	Positive
*cvaA*	Colicin V	Negative	Positive
*iss*	Increased serum survival protein	Positive	Negative
*sitA*	Iron transport protein	Positive	Positive
*ibeA*	Invasin	Negative	Positive
*vat*	Vacuolating autotransporter toxin	Negative	Positive
*ihA*-like	Putative Iha-like adhesin	Positive	Positive
*mchB*	Microcin	Positive	Positive
*mchC*	Microcin	Negative	Positive
*mchF*	Microcin	Negative	Positive
*mcmA*	Microcin	Negative	Positive
*gad*	Glutamate decarboxylase	Positive	Positive
*iha*	Adherence protein	Positive	Positive
*capU*	Hexosyltransferase homologue protein	Positive	Positive
*sigA*	Serine protease autotransporter	Positive	Positive
*stx1A-a*	Shiga toxin 1 variant a subunit A	Positive	Positive
*stx1B-a*	Shiga toxin 1 variant a subunit B	Positive	Positive
*kpsF*	Polysialic acid group 2 capsule expression protein	Positive	Positive
*kpsD*	Translocation of capsule of groups 2 and 3	Positive	Positive
*neuC*	K1 capsular antigen	Positive	Positive

*E. *coli* serotypes were O117:H7 (case 1, 8-day-old boy) and O156:H7 (case 2, 4-day-old boy). ST, sequence type.

Both strains differed substantially from *E.* *coli* K1 strains usually reported as causes of meningitis. K1 strains mainly belong to the ST95 complex (*7*) and most common serogroups are O18, O1, O7, O83, and O45 S88 (*6*,*8*). STEC O117:H7 ST504 strains were described in 2005 in 20 adults with persistent traveler’s diarrhea (*3*). Those strains and STEC strains described in another study of traveler’s diarrhea were atypical because they did not express lysine decarboxylase, β-galactosidase, intimin, or enterohemolysin (*4*). No invasive infections were reported before the 2 cases we describe, although some strains expressed extraintestinal virulence factors. However, because PCR for Stx is not routinely performed on neonatal invasive *E. coli* strains, STEC O117:H7 might be underestimated. Whole-genome sequencing of neonatal *E*. *coli* meningitis strains would help determine the role of STEC in fatal neonatal meningitis. In our patients, simultaneous presence of K1 antigen and salmochelin might explain isolate invasiveness (*1*). Moreover, STEC O156:H7 harbored the invasin IbeA, which promotes blood–brain barrier translocation.

When we analyzed the EnteroBase database (*9*) in late 2022, we identified only 4 O156:H7 and 39 O117:H7 strains distributed among 4 STs (ST504, n = 17; ST5292, n = 18; ST6880, n = 3; and ST9996, n = 1), all belonging to the ST504 complex. BLAST (https://blast.ncbi.nlm.nih.gov) analysis of STEC O117:H7 ST504 complex sequences from Enterobase consistently identified extraintestinal virulence factors yersinibactin and aerobactin (*5*); salmochelin (12 of 39 sequences) and K1 antigen (20 of 39 sequences) were inconsistently present. Since routine STEC sequencing began in 2017 in France, 3 other STEC O117:H7 ST504 complex strains have been identified (2 from patients with nonhemorrhagic diarrhea and 1 from an asymptomatic carrier).

Neither neonate described in this report had typical HUS, possibly because the *E*. *coli* strains lacked genes encoding Stx2, intimin, and enterohemolysin. However, Stx might have promoted intestinal translocation of the bacteria. Furthermore, antimicrobial drug therapy might have induced intracerebral Stx production, thereby contributing to fatal outcomes (*10*).

ST504 complex STEC strains exhibit only moderate intestinal virulence. However, we show that those strains can translocate into blood and CSF in neonates, especially if they produce K1 antigen and salmochelin. ST504-complex STEC expressing K1 antigen and salmochelin might be new hybrid pathotypes in neonates, even for those born at full term, with both extraintestinal pathogenic and neonatal meningitis virulence factors. Clinicians should remain aware of emerging pathotypes and new preventive and therapeutic strategies for *E*. *coli* infections in neonates.
